# The Role of a Biothesiometer in Early Detection and Management of Diabetic Neuropathy

**DOI:** 10.7759/cureus.70588

**Published:** 2024-10-01

**Authors:** Saili Kelshikar, Virendra Athavale, Rushabh A Parekh, Miten Ranka

**Affiliations:** 1 General Surgery, Dr. D. Y. Patil Medical College, Hospital and Research Centre, Dr. D. Y. Patil Vidyapeeth (Deemed to Be University), Pune, IND

**Keywords:** biothesiometer, diabetes mellitus, diabetic foot ulcers, diabetic peripheral neuropathy, vibration perception threshold

## Abstract

Introduction: Diabetic foot ulcers (DFUs) have become a common and serious outcome of diabetes mellitus (DM). Diabetic peripheral neuropathy (DPN) is often underdiagnosed, which impairs the capacity to prevent foot ulceration. The clinical manifestations typically include sensory complaints, which leave patients vulnerable to increased risk of injury without their awareness.

Aim: The current study aims to assess the viability of VPT (vibration perception threshold) testing using a biothesiometer as an early diagnostic tool for detecting diabetic neuropathy and reducing the burden of diabetic foot disease.

Material and methods: This prospective study was conducted in Dr. D. Y. Patil Medical College, Pimpri, Pune from August 2022 to July 2024 among 50 patients with DFUs. Ethical approval was taken from the Institutional Ethical Committee. An informed consent was obtained from all the study participants. VPT testing using a biothesiometer was done to assess the neurological status of the patient’s limbs. A significant difference between the symptomatic and asymptomatic cases was also calculated to evaluate the co-relation of symptoms compared to VPT.

Results: The largest age group among those surveyed is 41-50 with a mean age of 50.72. Males form a slightly larger proportion than females. A total of 22 (44%) ulcers are present over the plantar aspect of the foot. Out of the entire study, 37 (74%) individuals reported Grade II or III neuropathy, and 29 (58%) individuals reported no symptoms of diabetic neuropathy. The majority portion of individuals in the study had an HbA1c ranging from 6.5% to 8%. After an intervention, 44 (88%) patients did not experience a change in their VPT grading after six months.

Discussion: The chi-square test results suggest that the onset period of diabetes in years or the type of treatment history for diabetes does not significantly influence VPT grading among the surveyed individuals. However, individuals with higher HbA1c levels tend to exhibit more severe VPT impairments.

Conclusion: Regular monitoring of VPT grading is crucial for early diagnosis of neuropathy, tracking disease progression, and assessing intervention effectiveness. Hence, we recommend all diabetic patients regularly undergo biothesiometry as part of their routine diabetic check-ups to help prevent disease progression and complications like amputations and improve their overall quality of life.

## Introduction

Diabetes mellitus (DM) is a disorder marked by consistently elevated blood sugar levels. DM is categorized as type 1 and type 2 DM. While type 1 diabetes is characterized by a whole or near-total absence of insulin, type 2 DM encompasses a wide range of illnesses distinguished by reduced insulin production, different degrees of insulin resistance, increased glucose production, and modified fat metabolism. India has the second-highest number of diabetic patients, at 77 million, trailing behind China. The prevalence of type 2 DM in India is now 8.8% among those aged 20-79 years [1].

Diabetic foot syndrome, sometimes referred to as diabetic foot disease, comprises a range of medical disorders, such as peripheral vascular disease and diabetic peripheral neuropathy (DPN), that lead to the development of ulcers over the foot. Diabetic foot ulcers (DFUs) have become a common and serious outcome of DM [2]. DFUs have the potential to lead to amputation, especially in cases where there is wound infection or osteomyelitis [1,2].

Studies have indicated that those who have diabetes have a significant probability, as high as 25%, of having a foot ulcer at some point in their lives. DFUs are the main reason why people with diabetes need to be hospitalized, making up around 30% of all cases [3]. Moreover, the management of DFUs is costly. It accounts for 20% of the total costs in healthcare for diabetes, exceeding the spending related to any other diabetic illness [3]. The incidence of diabetic foot patients is rising in urban and rural India. Approximately 85% of amputations happen after the formation of foot ulcers. Around 75% of these amputations involve feet that are compromised by neuropathy and subsequent infection, a condition that might potentially be prevented. Neuropathic lesions are the cause of 80% of foot ulcers in India, whereas neuro-ischemic lesions are responsible for the remaining 20% [4].

DPN is often underdiagnosed, which impairs the capacity to prevent severe DPN and foot ulceration, diseases that have a substantial mortality risk within five years [5-7]. The clinical manifestations of DPN demonstrate considerable variety. Typically, sensory complaints are the primary characteristic, which leaves patients vulnerable to a higher risk of thermal and mechanical injury without their awareness. The progression from a minor injury to the formation of an ulcer, and ultimately the emergence of a persistent wound with an underlying infection, is commonly observed as the most prevalent series of events that precede the need for amputation of the lower leg [3].

The NICE (National Institute for Health and Care Excellence) recommendations recommend utilizing a 10 g Semmes-Weinstein monofilament and a 128 Hz tuning fork for conducting vibration perception tests in the screening of DPN. Adding VPT (vibration perception threshold) has greatly improved the early identification of peripheral neuropathy. Prior studies have shown that VPT is an effective technique for evaluating mild-to-moderate diabetic neuropathy [8]. VPT provides a quantifiable measure of the extent to which peripheral nerves are affected [9].

Hence, this study aims to assess the viability of VPT testing using a biothesiometer as an early diagnostic tool for detecting DPN and lowering the burden of diabetic foot disease.

## Materials and methods

This prospective study was conducted at Dr. D. Y. Patil Medical College, Hospital and Research Centre, Pimpri, Pune from August 2022 to July 2024. All patients with DM presenting with DFUs were included in the study. The sampling technique used was purposive sampling, with a sample size of 50 patients. The sample size calculation method was n=z^2^×P(100-P)/d^2^. Ethical approval was taken from the Institutional Ethical Committee (I.E.S.C/PGS/2022/73). An informed consent was obtained from all the study participants. Data was collected regarding demographics, detailed history, and physical examination, including a complete hemogram, blood sugar, and HbA1c levels. Clinical symptoms such as numbness, tingling, or burning sensation over the feet were noted.

VPT using a biothesiometer was done to assess the neurological status of the patient’s limbs at an average of 6 points in both feet - great toe, 1st, 3rd, and 5th metatarsal, instep, and heel. During the recording, the voltage was increased from 0 to 50 V. The grading is as follows: normal vibratory perception - 0 to 15 V; Grade I (mild loss of vibratory perception) - 16 to 20 V; Grade II (moderate loss of vibratory perception) - 21 to 25 V; Grade III (severe loss of vibratory perception) - above 25 V.

A significant difference between the symptomatic and asymptomatic cases was also calculated to evaluate the co-relation of symptoms compared to VPT. The cutoff value for significance was at p<0.05. Descriptive statistics were presented in the form of percentages. Statistical analysis was done using Epi-7 or open EPI (Centers for Disease Control and Prevention, Atlanta, GA). The statistical package used was WinPEPI (updated by J. H. Abramson).

Patients were given a printed report of their diabetic foot assessment with interpretation and asked to follow up for a repeat assessment after six months to check on the severity of their loss of vibration perception and peripheral neuropathy.

## Results

The largest age group among those surveyed is 41-50 years, comprising 24 (48%) of the total sample. The mean age of 50.72 years indicates the average age of the surveyed population. The study evaluated the outcomes of 50 individuals based on gender, with 27 males (54%) and 23 females (46%).

In Table 1, 22 (44%) ulcers are present over the plantar aspect of the foot. The ulcers on the heel predominantly exhibit Grade III VPT impairment, suggesting severe sensory loss in this area. The plantar aspect of the forefoot also shows a higher prevalence of severe impairment (Grade III), indicating significant sensory loss.

**Table 1:** Location of the ulcer with respect to different VPT gradings VPT, vibration perception threshold

Location of the ulcer	VPT grading				
	I, n (%)	II, n (%)	III, n (%)	Normal, n (%)	Total, n (%)
Dorsum of foot	3 (6)	5 (10)	3 (6)	4 (8)	15 (30)
Heel	0 (0)	0 (0)	8 (8)	0 (0)	8 (16)
Malleolus	2 (4)	4 (8)	5 (10)	2 (4)	13 (26)
Plantar aspect of the forefoot	1 (2)	3 (6)	9 (18)	1 (2)	14 (28)
Total	6 (12)	12 (24)	25 (50)	7 (14)	50 (100)

In Table 2, the chi-square test results suggest that the onset of diabetes (in years) does not significantly influence VPT grading among the surveyed individuals. This means that the distribution of VPT grades (I, II, III, and normal) does not vary considerably across different onset periods of diabetes.

**Table 2:** Association of the onset of diabetes in years with respect to different VPT gradings The chi-square statistic is 5.0132. The p-value is 0.83316. The result is not significant at p<0.05. VPT, vibration perception threshold

The onset of diabetes in years	VPT grading				
	I, n (%)	II, n (%)	III, n (%)	Normal, n (%)	Total, n (%)
0-2 years	1	2	1	1	5
3-5 year	2	3	9	1	15
6-8 years	2	5	12	4	23
9-11 years	1	2	1	0	4
12-14 years	0	0	1	1	2
15-17 years	0	0	1	0	1
Total	6	12	25	7	50
Mean	6.28				
Standard deviation	2.81				
Maximum	15				
Minimum	1				

In Table 3, 29 (58%) patients are on oral hypoglycemic agents (OHA) to treat diabetes. Among them, Grades II and III of neuropathy are most prevalent. The chi-square test results suggest that the type of treatment history (insulin, insulin + OHA, irregular, and OHA only) does not significantly influence VPT grading among the surveyed individuals.

**Table 3:** Association of treatment history for DM with respect to different VPT gradings The chi-square statistic is 2.9014. The p-value is 0.968077. The result is not significant at p<0.05. VPT, vibration perception threshold; DM, diabetes mellitus; OHA, oral hypoglycemic agents

Treatment history	VPT grading				
	I, n (%)	II, n (%)	III, n (%)	Normal, n (%)	Total, n (%)
Insulin	1 (2)	0 (0)	4 (8)	1 (2)	6 (12)
Insulin + OHA	0 (0)	0 (0)	3 (6)	2 (4)	5 (10)
Irregular	1 (2)	3 (6)	5 (10)	1 (2)	10 (20)
OHA	4 (8)	9 (18)	13 (26)	3 (6)	29 (58)
Total	6 (12)	12 (24)	25 (50)	7 (14)	50 (100)

In Table 4, 29 individuals (58%) reported no symptoms, with a relatively even distribution across VPT grades, the highest being Grade 3, loss of vibratory perception. Those individuals reporting a burning sensation predominantly exhibit Grade III VPT impairment. Individuals reporting numbness show a spread across VPT grades, with Grade II being the most prevalent. Similar to numbness, tingling also spreads across VPT grades, with Grade II being the most prevalent.

**Table 4:** Symptoms of diabetic neuropathy with respect to different VPT gradings VPT, vibration perception threshold

Symptoms of diabetic neuropathy	VPT grading				
	I, n (%)	II, n (%)	III, n (%)	Normal, n (%)	Total, n (%)
Burning sensation	0 (0)	0 (0)	4 (8)	0 (0)	4 (8)
None	2 (4)	4 (8)	16 (32)	7 (14)	29 (59)
Numbness	1 (2)	4 (8)	3 (6)	0 (0)	8 (16)
Tingling	3 (4)	4 (8)	2 (4)	0 (0)	9 (18)
Total	6 (12)	12 (24)	25 (50)	7 (14)	50 (100)

In Table 5, the chi-square test results suggest a statistically significant association between HbA1c levels and VPT grading among the surveyed individuals. Individuals with higher HbA1c levels (≥8% to <12.5%) tend to exhibit more severe VPT impairments (Grades II and III). Lower HbA1c levels (below the threshold of 8%) show minimal to no impairment (Grade I or normal).

**Table 5:** Association of HbA1c with respect to different VPT gradings The chi-square statistic is 13.6302. The p-value is 0.034051. The result is significant at p<0.05. VPT, vibration perception threshold

HbA1c	VPT grading				
	I, n (%)	II, n (%)	III, n (%)	Normal, n (%)	Total, n (%)
Normal (<5.7%)	0 (0)	0 (0)	0 (0)	0 (0)	0 (0)
Prediabetes (5.7%-6.4%)	0 (0)	0 (0)	0 (0)	0 (0)	0 (0)
Diabetes (>6.5%-<8)	5 (10)	5 (10)	4 (8)	6 (12)	20 (40)
Diabetes (≥8% to <10%)	1 (2)	5 (10)	12 (24)	0 (0)	18 (36)
Diabetes (≥10% to <12.5)	0 (0)	2 (4)	9 (18)	1 (2)	12 (24)
Total	6 (12)	12 (24)	25 (50)	7 (14)	50 (100)
Mean	8.852				
Standard deviation	1.64				
Maximum	12.2				
Minimum	6.7				

In Table 6, 12 (24%) individuals with Grade III VPT impairment received the intervention of diabetic control with footwear with a smaller proportion receiving dressings with footwear as an intervention. Individuals with Grade II VPT impairment mostly received medical management with footwear as intervention followed by surgical intervention with footwear.

**Table 6:** Intervention with respect to different VPT gradings VPT, vibration perception threshold

Intervention	VPT grading				
	I, n (%)	II, n (%)	III, n (%)	Normal, n (%)	Total, n (%)
Diabetic control + footwear	0 (0)	0 (0)	14 (24)	2 (4)	16 (34)
Dressings + footwear	1 (2)	2 (4)	7 (14)	2 (4)	12 (24)
Medical management + footwear	2 (4)	6 (12)	1 (2)	0 (0)	9 (18)
Surgical intervention + footwear	3 (6)	4 (8)	3 (6)	3 (6)	13 (23)
Total	6 (12)	12 (24)	25 (50)	7 (14)	50 (100)

In Table 7, all of the individuals with Grade III VPT impairment showed an unchanged outcome in VPT after six months. Five (10%) individuals with Grade II VPT impairment and 1 (2%) with Grade I VPT impairment showed deteriorated VPT grading after six months. Overall, 44 (88%) individuals did not experience a change in their VPT grading after six months.

**Table 7:** Outcome after six months with respect to different VPT gradings VPT, vibration perception threshold

Outcome after six months	VPT grading				
	I, n (%)	II, n (%)	III, n (%)	Normal, n (%)	Total, n (%)
Deteriorated	1 (2)	5 (10)	0 (0)	0 (0)	6 (12)
Unchanged	5 (10)	7 (14)	25 (50)	7 (14)	44 (88)
Total	6 (12)	12 (24)	25 (50)	7 (14)	50 (100)

## Discussion

After the data analysis, it was found that 24 (48%) participants were between 41 and 50 years old. The gender distribution indicates a relatively balanced sample with a slight majority of males. This balance suggests that the survey population is representative of gender, allowing for insights into both male and female perspectives. According to a study by Young et al., patients were more likely to develop foot ulcers if they had a baseline VPT threshold above 25 volts when evaluated with the biothesiometer. In this study, Grade III neuropathies were seen in 40 male patients and 39 female patients, which signifies that the neuropathy severity was not affected by gender. However, the age showed a correlation with peripheral neuropathy. Sixty patients who were more than 50 years of age showed Grade III neuropathies. Nineteen patients who were less than 50 years of age scored Grade III neuropathy [10]. This finding corroborates with our study findings wherein 16 (32%) patients above the age of 45 years showed Grade III neuropathy when evaluated with a biothesiometer and nine (18%) patients below the age of 45 years showed Grade III neuropathy.

In our present study, 22 (44%) ulcers are prevalent over the plantar aspect of the foot. Grade III VPT impairment is seen in ulcers over the heel and the metatarsal heads suggesting severe sensory loss in this area. In a study by Patil et al. in 2018, most DFUs were present on the plantar aspect of the foot, 38% on metatarsal heads, and 18% over the heels with 60.4% of the total having severe VPT impairment, which corroborates with our study where 14 (28%) ulcers were present over the plantar aspect of the forefoot and eight (16%) over the heel of the foot with 25 (50%) of the total having Grade III loss of vibratory perception [11].

In the current study, the most frequent range of onset of diabetes is six to eight years. The chi-square test results suggest that the onset of diabetes does not significantly determine VPT grading among the surveyed individuals. However, the study conducted by Young et al. evaluated VPT as an anticipator of foot ulceration risk in diabetes patients. In that study, patients who had VPT less than 15 V had a lower prevalence of foot ulcers (2.9%) when set side by side with those with VPT more than 25 V (19.8%). The likelihood of developing foot ulcers increases with the duration of diabetes. Even after adjusting for diabetes duration, patients with higher VPT still had a significantly higher risk of ulceration. Utilizing VPT measurements can help recognize patients who have an increased susceptibility to foot ulcerations, allowing for targeted foot-care education and interventions [10].

The chi-square test results in our present study suggest that the type of treatment history (insulin, insulin + OHA, irregular, and OHA only) does not significantly influence VPT grading among the surveyed individuals. In the study conducted by Jayaprakash et al. in 2011, 42.2% were taking insulin and/or oral antidiabetic drugs and 34.9% showed prevalence of peripheral neuropathy in their study sample with a biothesiometer [12]. Likewise, a study by Lazo Mde et al. in 2014 reported that those with more than 10 years of diabetes and patients taking OHA with insulin were 40% more likely to have neuropathy [13]. These results indicate a higher risk of peripheral neuropathy in people with more advanced diabetes who need more than just one treatment for diabetes. This does not match with our present findings of most patients being on oral hypoglycemic agents. This is probably because our sample size is small.

In our study, the biothesiometer is used as the standard method for the detection of DPN. Twenty-nine (58%) patients gave no symptoms of neuropathy; however, on evaluation with a biothesiometer, 38 (74%) patients (this includes the patients with a history of burning sensation, tingling, and numbness) had Grade II or III neuropathy. Twenty-five (50%) of the total patients had Grade III loss of vibratory perception when evaluated with a biothesiometer whereas 12 (24%) showed Grade II neuropathy. Only seven (14%) patients had no loss of vibratory perception, all of whom reported no symptoms of diabetic neuropathy. Individuals reporting numbness and tingling show a spread across VPT grades, with Grade II being the most prevalent. Those reporting a burning sensation predominantly exhibit Grade III VPT impairment. The sample size in our study is only 50, but the objective was to detect neuropathy in patients and educate them on foot care. The present study results correlated with Aruna and Haragopal who in 2017 stated a statistically significant difference in the mean VPT among diabetic and non-diabetic groups, which concluded that all patients with diabetes regardless of neuropathic symptoms must undergo regular biothesiometry as part of their routine diabetic checkup. This may help in the early diagnosis of peripheral neuropathy and further treatment planning to avert complications [14]. A similar study by Devi et al. in 2022 showed 85 patients who gave no symptoms of neuropathy, but on biothesiometry, out of 100, 91 had sensory neuropathy of Grade II or III, which supports our study wherein 29 (58%) patients gave no symptoms of neuropathy, but on evaluation, 37 (74%) had Grade II or III neuropathy [15].

In the present study, a significant chi-square result suggests that HbA1c levels are associated with VPT grading among the surveyed individuals. This association underscores the importance of glycemic control in managing diabetic neuropathy, as higher HbA1c levels indicate poorer sensory nerve function. A study conducted by Ramchandra et al. evaluated the usefulness of a biothesiometer by assessing peripheral neuropathy in non-insulin-dependent diabetic patients. The readings were significantly improved after the control of hyperglycemia. Biothesiometry is a sensitive index to quantify the threshold of vibratory sensation. With the correction of blood glucose levels, the biothesiometer readings can change just as seen in nerve conduction velocities [16]. A similar study by Jian-bin Su et al. in 2018 concluded that HbA1c can be considered a strong indicator of DPN. Increased glycated hemoglobin levels are closely associated with peripheral neuropathy in type 2 DM patients [17].

In our study, individuals received a variety of treatments like dressings, diabetic control, surgical interventions like debridement and amputation, and medical management to improve the ulcers. Forty-four (88%) individuals did not experience a change in their VPT grading after six months.

Treatment of DFUs included removal of callus and dead tissue, good wound care, blood glucose control, nutrition, antibiotics, vascular evaluation, and pressure offloading. The offloading helps to relieve abnormal pressure that is applied to the ulcer and promotes healing of the wound. Methods like felted foam dressings, total contact casting (TCC), short-leg walkers, and half-shoes have been applied for offloading [18].

All individuals in our study were advised appropriate diabetic footwear. Diabetic footwear must be used by all diabetic patients after consulting a podiatrist. The shoes must provide adequate cushioning and support. They must be made of comfortable material and must be properly fitting to prevent calluses [18]. Patient education remains an integral part of the management of DFUs. Therefore, we advise diabetic patients to include biothesiometry as a part of their regular diabetic check-ups. This can aid in the timely detection of DPN and allow for the planning of further treatment to prevent the disease from advancing, which could otherwise lead to diabetic ulcer formation. A multidisciplinary team approach is required to provide a successful plan for the prevention and treatment of diabetic foot disease. This includes a diabetologist/endocrinologist to optimize the metabolic control of diabetic patients, a qualified nurse or diabetes educator, a podiatrist that guides the patient and provides appropriate treatment to prevent foot lesions, an orthotist to suggest appropriate footwear, a vascular surgeon to assess the lower limb vascularity and give necessary interventional management, a nutritional consultant that helps in glycemic control and weight loss, and an infection disease specialist to start appropriate antibiotics based on the culture sensitivity report [18].

Limitations

Our study had several limitations. First, the sample size was small. Second, a longer observation period would be necessary to understand the long-term outcomes and potential recurrence of DFUs following the treatments. Additionally, it may have been beneficial to include diabetic patients without foot ulcers to detect subclinical DPN. Finally, the VPT assessment using a biothesiometer was done by a single individual, which may have introduced bias and affected the reliability of our results. Further studies are needed to address these limitations and provide a more comprehensive evaluation of the interventions.

## Conclusions

Within the limitations of our present study, we conclude that higher HbA1c levels and specific ulcer locations contribute to more severe sensory impairments, highlighting the importance of glycemic control and targeted interventions. Regular monitoring of VPT grading is crucial for early diagnosis of neuropathy, tracking disease progression, and assessing intervention effectiveness. Hence, we recommend that all diabetic patients regularly undergo biothesiometry as part of their routine diabetic check-ups. This approach may help prevent the progress of disease, and complications like amputations and improve overall quality of life.

## References

[1] NCD Risk Factor Collaboration (2016). Worldwide trends in diabetes since 1980: a pooled analysis of 751 population-based studies with 4.4 million participants. Lancet.

[2] Wild S, Roglic G, Green A, Sicree R, King H (2004). Global prevalence of diabetes: estimates for the year 2000 and projections for 2030. Diabetes Care.

[3] Tesfaye S, Boulton AJ, Dyck PJ (2010). Diabetic neuropathies: update on definitions, diagnostic criteria, estimation of severity, and treatments. Diabetes Care.

[4] Bansal V, Kalita J, Misra UK (2006). Diabetic neuropathy. Postgrad Med J.

[5] Nabuurs-Franssen MH, Houben AJ, Tooke JE, Schaper NC (2002). The effect of polyneuropathy on foot microcirculation in Type II diabetes. Diabetologia.

[6] England JD, Gronseth GS, Franklin G (2005). Distal symmetric polyneuropathy: a definition for clinical research: report of the American Academy of Neurology, the American Association of Electrodiagnostic Medicine, and the American Academy of Physical Medicine and Rehabilitation. Neurol.

[7] Didangelos T, Doupis J, Veves A (2014). Painful diabetic neuropathy: clinical aspects. Handb Clin Neurol.

[8] Boulton AJ, Vinik AI, Arezzo JC (2005). Diabetic neuropathies: a statement by the American Diabetes Association. Diabetes Care.

[9] Saha D, Saha K, Dasgupta PK (2010). Vibration sense impairment in diabetes mellitus. Indian J Physiol Pharmacol.

[10] Young MJ, Breddy JL, Veves A, Boulton AJ (1994). The prediction of diabetic neuropathic foot ulceration using vibration perception thresholds. A prospective study. Diabetes Care.

[11] Patil A, More D, Patil A, Jadhav KA, Vijil Mejia ME, Patil SS (2018). Clinical, etiological, anatomical, and bacteriological study of “diabetic foot” patients: results of a single center study. Cureus.

[12] Jayaprakash P, Bhansali A, Bhansali S, Dutta P, Anantharaman R, Shanmugasundar G, Ravikiran M (2011). Validation of bedside methods in evaluation of diabetic peripheral neuropathy. Indian J Med Res.

[13] Lazo Mde L, Bernabé-Ortiz A, Pinto ME (2014). Diabetic peripheral neuropathy in ambulatory patients with type 2 diabetes in a general hospital in a middle income country: a cross-sectional study. PLoS One.

[14] Aruna B, Haragopal R (2017). Role of Biothesiometry in the diagnosis of diabetic neuropathy. Indian J Clin Anat Physiol.

[15] Devi M, Singh S, Dhanawat M, Gupta K, Gupta S, Agarwal BK (2022). Assessment of peripheral neuropathy pain by biothesiometer in diabetes mellitus patients. J Young Pharm.

[16] Ramachandran A, Mohan V, McMillan DE, Snehalatha C, Chinnikrishnudu M, Viswanathan M (1987). Evaluation of clinical neuropathy in diabetes: use and limitations of biothesiometer. J Diabetic Assoc India.

[17] Su JB, Zhao LH, Zhang XL (2018). HbA1c variability and diabetic peripheral neuropathy in type 2 diabetic patients. Cardiovasc Diabetol.

[18] Amin N, Doupis J (2016). Diabetic foot disease: from the evaluation of the "foot at risk" to the novel diabetic ulcer treatment modalities. World J Diabetes.

